# Circulating MicroRNAs in Patients with Chronic Hepatitis C and Non-Alcoholic Fatty Liver Disease

**DOI:** 10.1371/journal.pone.0023937

**Published:** 2011-08-23

**Authors:** Silvia Cermelli, Anna Ruggieri, Jorge A. Marrero, George N. Ioannou, Laura Beretta

**Affiliations:** 1 Public Health Sciences Division, Fred Hutchinson Cancer Research Center, Seattle, Washington, United States of America; 2 Department of Infectious, Parasitic and Immune-Mediated Disease, Istituto Superiore di Sanità, Roma, Italy; 3 Division of Gastroenterology, Department of Internal Medicine, University of Michigan, Ann Arbor, Michigan, United States of America; 4 Division of Gastroenterology, Department of Medicine, Veterans Affairs Puget Sound Health Care System and University of Washington, Seattle, Washington, United States of America; Saint Louis University, United States of America

## Abstract

MicroRNAs miR-122, miR-34a, miR-16 and miR-21 are commonly deregulated in liver fibrosis and hepatocellular carcinoma. This study examined whether circulating levels of these miRNAs correlate with hepatic histological disease severity in patients with chronic hepatitis C infection (CHC) or non-alcoholic fatty-liver disease (NAFLD) and can potentially serve as circulating markers for disease stage assessment. We first used an *in vitro* model of hepatitis C virus (HCV) infection to measure the extracellular levels of these four miRNAs. Whereas miR-21 extracellular levels were unchanged, extracellular levels of miR-122, miR-34a and to a lesser extent miR-16, steadily increased during the course of HCV infection, independently of viral replication and production. Similarly, in CHC patients, serum levels of miR-122, miR-34a and miR-16 were significantly higher than in control individuals, while miR-21 levels were unchanged. There was no correlation between the serum levels of any of these microRNAs and HCV viral loads. In contrast, miR-122 and miR-34a levels positively correlated with disease severity. Identical results were obtained in an independent cohort of CHC patients. We extended the study to patients with NAFLD. As observed in CHC patients, serum levels of miR-122, miR-34a and miR-16 were significantly higher in NAFLD patients than in controls, while miR-21 levels were unchanged. Again, miR-122 and miR-34a levels positively correlated with disease severity from simple steatosis to steatohepatitis. In both CHC and NAFLD patient groups, serum levels of miR-122 and miR-34a correlated with liver enzymes levels, fibrosis stage and inflammation activity. miR-122 levels also correlated with serum lipids in NAFLD patients. Conclusion: Serum levels of miR-34a and miR-122 may represent novel, noninvasive biomarkers of diagnosis and histological disease severity in patients with CHC or NAFLD.

## Introduction

Liver biopsy is often recommended in patients with unexplained elevated serum aminotransferases in order to determine the cause, to stage hepatic fibrosis and to grade hepatic inflammation. Non-invasive methods that can evaluate disease severity and the likelihood of disease progression in persons with elevated liver enzymes need to be developed. MicroRNAs are small non-coding RNAs that control translation and transcription of many genes. They are receiving growing attention because of numerous reports on their dysregulation in human diseases and their potential as diagnostic and therapeutic targets. Because of their stability and presence in almost all body fluids, miRNAs constitute a novel class of non-invasive biomarkers. Numerous studies have shown that aberrant miRNA expression is associated with the development and progression of various types of human cancer and therefore studies on circulating miRNA profiles largely focused on cancer [1]–[3].

This study examined whether serum levels of selected miRNAs, thought to be deregulated in liver disease, can serve as non-invasive biomarkers of diagnosis and histological severity in patients with chronic hepatitis C (CHC) or non-alcoholic fatty liver disease (NAFLD). The highly abundant liver-specific miR-122, is of particular interest. miR-122 is known to regulate metabolic pathways in the liver, including cholesterol biosynthesis [4]–[7]. miR-122 also positively regulates hepatitis C virus (HCV) replication and viral production [8]–[10]. Reduced expression of miR-122 has been observed in hepatocellular carcinoma (HCC), often in advanced tumors of poor prognosis [11]–[13] although an upregulation of miR-122 was also reported in HCV-derived HCC [11], [14]. miRNAs encoded by the miR-15/16 cluster act as tumor suppressors and are down-regulated in several human cancers [15]. In contrast, miR-21 was identified to be consistently upregulated in many cancers [16] including HCC [17]. Upregulation of miR-21 was also found in highly fibrotic HCV-infected human livers [18]. miR-34a, a central mediator of p53 function [19], has recently emerged as another miRNA modulated in liver disease. Interestingly, while most studies report a downregulation of miR-34a in human cancers [19], miR-34a was found increased in HCC [17] as well as in a mouse model of steatohepatitis [20]. We selected these four miRNAs (miR-122, miR-16, miR-21 and miR-34a) for the present study aimed at investigating their levels in serum of patients with CHC and NAFLD with a wide spectrum of histological disease severity. In addition to this analysis on human sera, we used an *in vitro* model of HCV infection to measure extracellular levels of these same miRNAs in supernatant of HCV-infected cells.

## Materials and Methods

### Ethics Statement

The study protocol conforms fully to the ethical guidelines of the 1975 Declaration of Helsinki and was approved by the Institutional Review Board of the Fred Hutchinson Cancer Research Center. We used only fully de-identified samples that were transferred from formal sample repositories at the University of Michigan and the Veterans Affairs Puget Sound Health Care System. Written, informed consent was obtained from every human subject when the samples were originally collected for the repositories under the terms of study protocols approved by the Institutional Review Boards at these respective institutions.

### *In vitro* HCV infection and HCV RNA quantitation

HCV viral stocks were generated following transfection of *in vitro* transcripts of the HCVJ6/JFH genotype 2a strain (kindly provided by Dr. Charles Rice) using DMRIE-C (Invitrogen). Huh7.5 cells were infected with HCVcc, at a dose of 5.6×10^3^ TCID_50_/ml as described [21] and cultured for 15 days, corresponding to 4 passages. Cells and supernatants were collected at five, ten and fifteen days post-infection, 48 hrs after the cells were split and seeded at a density of 2×10^5^ cells/cm^2^. The confluence of the cells at the time of collection was 70-80%. Intracellular and extracellular RNA was extracted using the miRNeasy extraction kit (QIAGEN). Samples were submitted to DNAse digestion, reverse-transcription using random hexamers, and real-time PCR using the following HCV primer sequences: 5-CGGGAGAGCCATAGTGGTCTGCG-3 and 5-CTCGCAAGCACCCTATCAGGCAGTA-3. To determine HCV copy numbers, standard curves were prepared by serial dilution of a plasmid bearing the amplified HCV sequence.

### Patient Groups (Table S1)

The Control group was composed of 19 healthy individuals without any evidence of liver disease. The Chronic Hepatitis C (CHC) group was composed of a first set of 18 patients recruited at the University of Michigan and of an independent set of 35 patients recruited as part of an ongoing observational study of chronic liver disease at Veterans Affairs Puget Sound Health Care System. HCV infection was defined by presence of HCV RNA in serum. The Non-Alcoholic Fatty Liver Disease (NAFLD) group was composed of 34 patients also enrolled at Veterans Affairs Puget Sound Health Care System. NAFLD was defined by the presence of hepatic steatosis in at least 5% of hepatocytes, in the absence of HCV RNA and hepatitis B virus surface antigen, self-reported alcohol consumption in the preceding six months, or histological features suggestive of primary biliary cirrhosis, autoimmune hepatitis, or iron overload.

For hepatic histology assessment, formalin-fixed liver tissue was stained with hematoxylin and eosin, Masson's trichrome and special stains for iron and copper and reviewed independently by a liver pathologist, who was blinded to this study. For CHC patients, the Batts and Ludwig scoring system [22] was used to score fibrosis (0–4) and inflammation (0–4). For NAFLD patients, steatosis, ballooning degeneration, inflammation, and fibrosis were scored according to the currently accepted scoring system [23]. In this scoring system, the scores for steatosis grade (0–3), lobular inflammation (0–3), and ballooning (0–2) can be summed to yield a NAFLD Activity Score (NAS) with scores ≥5 being considered diagnostic for histological steatohepatitis while scores of 1–4 are diagnostic of simple steatosis.

### miRNA quantitation

Total RNA with preserved miRNAs was extracted from 100 µl of serum by miRNeasy extraction kit and using a plasma/QIAzol ratio of 1∶10. Synthetic spiked-in *C. elegans* miR-238 was added to the serum and cell supernatant samples prior to RNA extraction as internal control and RNU44 was quantified in the cellular RNA samples. Expression of mature miRNAs was detected using the Taqman miRNA qRT-PCR Assay (Applied Biosystems, Carlsbad, CA). Reverse transcription and PCR reactions were run in triplicate in the Applied Biosystems 7900 System. To determine miRNA copy numbers, standard curves were prepared by serial dilution of synthetic miRNA (Integrated DNA Technologies, Coralville, IA).

### Statistical Analysis

Statistical significance of differences between groups was analyzed by Wilcoxon rank sum test using TIBCO Spotfire S+ (TIBCO Spotfire, Somerville, MA). Correlation analysis was performed using two-tailed Pearson correlation test. Receiver operating characteristic (ROC) analysis was undertaken using R software version 2.9.2.

## Results

### Increased levels of miR-122, miR-34a and miR-16 in the supernatant of HCV-infected cells

**Figure 1:**
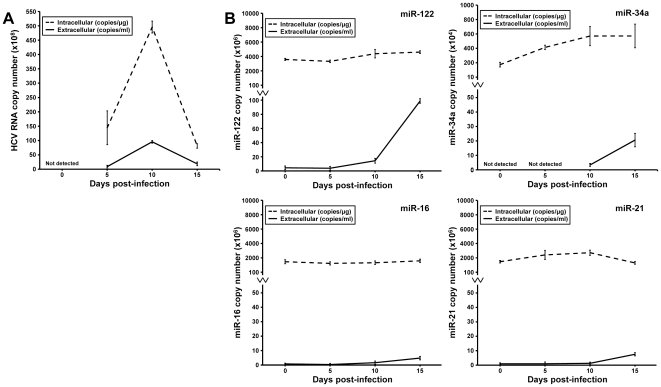
Differential expression of miRNAs in supernatant of HCV-infected cells. (A) Detection of intracellular and extracellular HCV RNA. (B) miR-122, miR-34a, miR-16 and miR-21 levels in uninfected and HCV-infected Huh7.5 cells at 5, 10 and 15 days post-infection. Cycle threshold (C_T_) values were converted to an absolute value based on the standard curve. The expression levels are presented as the mean ± standard error of the mean (SEM) (copy number/ml of supernatant or copy number/µg of total RNA) of three independent experiments.

Intracellular and extracellular levels of miR-122, miR-34a, miR-16 and miR-21 were measured in Huh7.5 cells infected with a genotype 2a chimeric HCV, after confirming the absence of confounding miRNAs in the bovine serum used to culture the cells. Copy numbers of the targeted miRNAs and of the HCV RNA were calculated at five, ten and fifteen days post-infection in three independent experiments. HCV RNA reached the highest levels at 10 days post-infection in both the intracellular and extracellular compartments (4.9×10^10^ copies/µg and 0.9×10^10^ copies/ml, respectively) (Figure 1A). Among the four miRNAs analyzed, miR-122 was the most abundant in both the intracellular and extracellular compartments (3.7×10^9^ copies/µg of total RNA and 4.4×10^6^ copies/ml of supernatant, respectively) and miR-34a the least abundant with 1.8×10^6^ intracellular copies/µg of total RNA and undetectable extracellular levels (Figure 1B). miR-16 and miR-21 abundances were largely similar with 1.6×10^9^ intracellular copies/µg of total RNA for both miRNAs, 7.6×10^5^ copies/ml for extracellular miR-16 and 9×10^5^ copies/ml of supernatant for extracellular miR-21 (Figure 1B). Intracellular levels of miR-122, miR-16 and miR-21 remained constant during the infection while intracellular miR-34a levels increased by 2.3-fold at 5 days post-infection and by 3.2-fold at 10 and 15 days post-infection (Figure 1B). At 10 days post-infection, corresponding to the highest levels of HCV RNA, extracellular levels of miR-122 and miR-16 increased by 3.3-fold and 2.1-fold, respectively, while miR-21 remained unchanged. miR-34a levels were undetectable in supernatant of uninfected Huh7.5 cells as well as in cells infected with HCV for 5 days, but increased to detectable levels at 10 days post-infection. At 15 days post-infection, corresponding to a decline in HCV RNA levels, extracellular miR-122, miR-34a and miR-16 levels further increased by 6.9-, 6.2- and 2.9-fold, respectively and a 6-fold accumulation of miR-21 was observed (Figure 1B).

### Increased serum levels of miR-122, miR-34a and miR-16 in chronic hepatitis C patients

**Figure 2:**
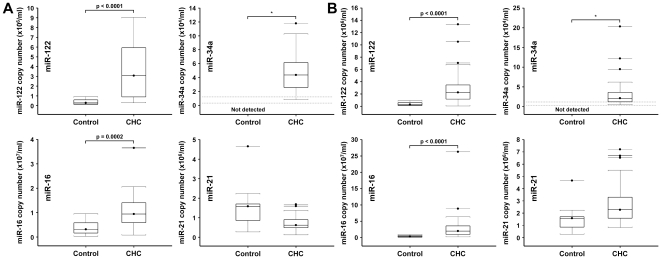
Up-regulation of serum miR-122, miR-34a and miR-16 in chronic hepatitis C patients. Serum levels of miR-122, miR-34a, miR-16 and miR-21 in (A) a first set of 18 CHC patients and (B) an independent set of 35 CHC patients. C_T_ values were converted to an absolute value based on the standard curves. Serum miRNA expression levels are expressed in copy number/ml. In the box-plot displays, the bold line indicates the median per group, the box represents 50% of the values and horizontal lines show minimum and maximum values of the calculated non-outlier values. For miR-34a, the dashed lines represent the levels corresponding to C_T_ values between 35 and 37 (1.2×10^4^ – 0.3×10^4^copies/ml).

We then investigated whether these miRNAs are detected and modulated in serum from patients with chronic hepatitis C (CHC). In healthy control sera, levels of miR-16 (3.1×10^6^ copies/ml) were higher than levels of miR-21 (1.6×10^6^ copies/ml) and miR-122 (2.8×10^5^ copies/ml), and miR-34a was undetectable, the detection limit of the assay being approximately 0.3×10^4^ copies/ml. Serum levels of miR-122 and miR-16 were significantly higher in a first set of patients with CHC (n = 18) compared to healthy controls (n = 19) (10.8-fold (p<0.0001) and 3.0-fold (p = 0.0002), respectively) and miR-34a levels increased from undetectable levels to a median level of 4.4×10^4^ copies/ml (Figure 2A). miR-21 levels were not significantly different between controls and CHC patients (Figure 2A). There was no correlation between the abundance of any of these four miRNAs and the HCV viral load measured in these patients (R from -0.08 to 0.08). We validated these results in an independent group of 35 CHC patients enrolled at a different site. Confirming the results obtained in the first set of patients, miR-122 levels were higher by 7.9-fold in CHC patients compared to controls (p<0.0001), miR-16 levels were higher by 6.3-fold (p<0.0001), miR-34a was undetectable in control sera but detected at a median of 2.1×10^4^ copies/ml in the CHC group, and miR-21 levels were unchanged (Figure 2B). Finally, there was no correlation between the serum abundance of any of these four miRNAs and the HCV viral load (R from -0.003 to 0.24).

**Figure 3:**
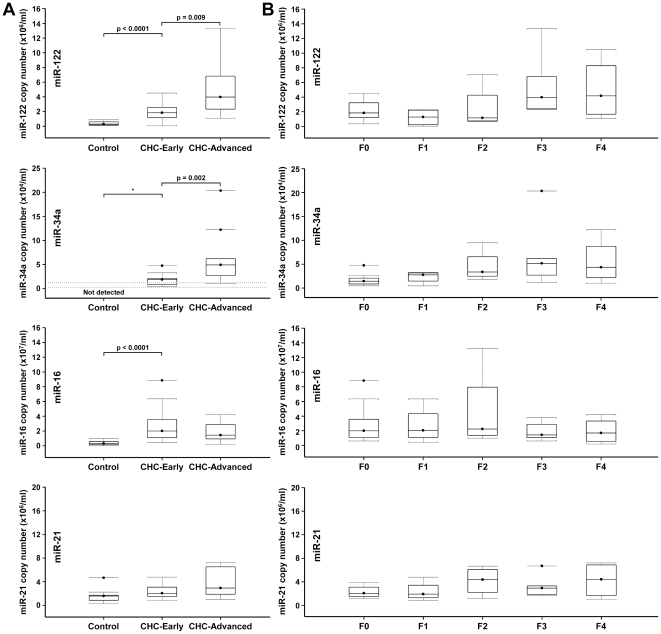
Serum levels of miR-122, miR-34a, miR-16 and miR-21 and histological liver disease severity in CHC patients. CHC group was subdivided into (A) CHC-early (F0-F1) and CHC-advanced groups (F3-F4) and (B) further subdivided according to individual fibrosis stage.

Based on histology grading, we separated the CHC patients into patients with early stage fibrosis (F0-F1) and patients with advanced fibrosis (F3-F4). miR-122 and miR-16 serum levels were already strongly increased in the CHC-early group compared to controls (6.4-fold and p<0.0001 for both miRNAs) and miR-34a levels were detectable in all patients with early disease, with a median of 1.8×10^4^ copies/ml (Figure 3A). Serum levels of miR-122 and miR-34a further increased in the CHC-advanced group compared to the CHC-early group (2.2-fold (p = 0.009) and 2.6-fold (p = 0.002), respectively) (Figure 3A). The increase of miR-122 mostly occurred between F2 and F3 stages while the increase of miR-34a was continuous between F0–F3 (Figure 3B). In contrast to miR-122 and miR-34a, miR-16 serum levels remained unchanged in the CHC-advanced group compared to the CHC-early group (Figure 3A). There was no significant difference in miR-21 levels between the CHC-early and CHC–advanced groups although a slight increase was observed in patients with F4 stage disease (Figure 3A,B).

### Increased serum levels of miR-122, miR-34a and miR-16 in patients with NAFLD

**Figure 4:**
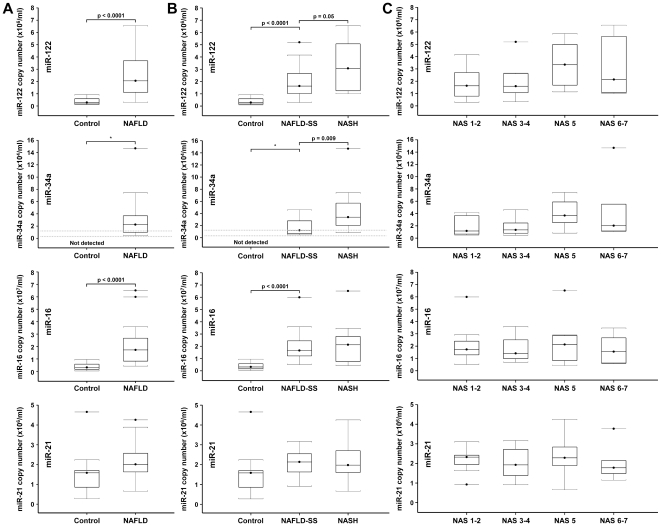
Serum levels of miR-122, miR-34a, miR-16 and miR-21 in NAFLD patients. (A) Expression of miR-122, miR-34a, miR-16 and miR-21 in serum from healthy controls and NAFLD patients. (B) NAFLD patients were divided into two groups based on the NAFLD activity score (NAS): NAFLD-simple steatosis (NAFLD-SS) with NAS≤4 and non-alcoholic steatohepatitis (NASH) with NAS ≥5. (C) NAFLD-SS and NASH groups were further subdivided based on individual NAS scores.

To determine whether serum levels of miR-122, miR-34a, miR-16 and miR-21 change in patients with other chronic liver diseases, we measured these four miRNAs in sera collected from 34 patients diagnosed with NAFLD. miR-122 levels were increased by 7.2-fold in NAFLD patients compared to healthy controls (p<0.0001), miR-16 levels were increased by 5.5-fold (p<0.0001), miR-34a increased from undetectable levels to a median of 2.2×10^4^ copies/ml and miR-21 was unchanged (Figure 4A). We divided the NAFLD patients into two groups based on the NAFLD activity score (NAS): patients with simple steatosis (NAFLD-SS) defined by NAS scores 1–4 and patients with non-alcoholic steatohepatitis (NASH) defined by NAS scores 5–7. Serum levels of miR-122 and miR-16 were higher in NAFLD-SS compared to the controls (5.7-fold (p<0.0001) and 5.3-fold (p<0.0001), respectively) and miR-34a were detectable in all patients with simple steatosis with a median of 1.2×10^4^ copies/ml. miR-21 levels were unchanged. miR-122 and miR-34a levels further increased in the NASH group compared to the NAFLD-SS group (2.0-fold (p = 0.05) and 2.8-fold (p = 0.009), respectively) (Figure 4B). For both miRNAs, the increase occurred mostly between NAS scores 3-4 and NAS score 5 (Figure 4C). miR-16 and miR-21 levels were similar in both groups (Figure 4B).

### miR-122 and miR-34a correlation with clinical parameters and performance in disease stage assessment

Because both miR-122 and miR-34a levels correlate with disease severity, we investigated the relationship of their serum levels with the clinicopathological parameters collected from the CHC and NAFLD patients (Table 1). In both patient groups, a strong positive correlation with alanine aminotransferase (ALT) and aspartate aminotransferase (AST) was observed for miR-122. High positive correlations were also observed for miR-122 and miR-34a with fibrosis stage and inflammation activity. In CHC patients, a positive correlation was also observed for miR-122 and miR-34a with blood glucose levels and insulin resistance. In NAFLD patients, a positive correlation was also observed between miR-122 and total cholesterol level and low-density lipoprotein (LDL).

**Table 1:** Correlation between miRNAs and specific clinical parameters.

	CHC			NAFLD		
		miR-122	mir-34a		miR-122	mir-34a
ALT (IU/L)	59 (26–394)*	0.92	0.86	76 (14–183)*	0.75	0.45
AST (IU/L)	47 (23–336)*	0.88	0.85	41.5 (15–141)*	0.55	0.46
NAS score: ≤4/≥5	NA	NA	NA	53/47**	0.38	0.46
Fibrosis stage: ≤1/≥2	60/40**	0.50	0.51	79/21**	0.33	0.41
Inflammation activity: ≤1/≥2	62/38**	0.50	0.50	62/38**	0.33	0.43
Steatosis grade: ≤1/≥2	91/9**	0.55	0.58	47/53**	0.11	0.13
Balloon Hepatocytes: ≤1/≥2	NA	NA	NA	73/27**	0.46	0.43
Total Cholesterol (mg/dL)	177 (104–274)*	−0.16	−0.10	204.5 (141–288)*	0.36	0.11
LDL (mg/dL)	107 (46–195)*	−0.09	−0.03	113 (43–182)*	0.44	0.19
HDL (mg/dL)	46 (27–74)*	−0.24	−0.34	40 (25–74)*	0.25	0.24
Triglycerides (mg/dL)	104 (51–245)*	0.10	0.20	222.5 (75–879)*	−0.14	−0.18
Glucose (mg/dL)	104 (60–292)*	0.45	0.42	111 (77–302)*	−0.19	−0.21
Insulin resist. (HOMA score)	3.5 (0.6–12.2)*	0.39	0.34	6.6 (1.4–89.9)*	0.09	0.13

*median (range); ** (%).

**Figure 5:**
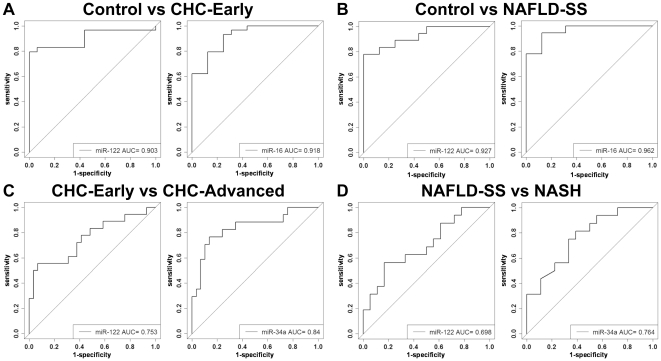
Receiver operating characteristic (ROC) analysis of expression of miRNAs in CHC and NAFLD patients. ROC curves with corresponding area under the ROC curve (AUC) for (A): mir-122 and miR-16 from CHC-early versus controls; (B) mir-122 and miR-16 from NAFLD-SS (NAS 1-4) versus controls; (C) miR-122 and miR-34a from CHC-early versus CHC-advanced; (D) miR-122 and miR-34a from NAFLD-SS(NAS 1-4) versus NASH (NAS 5–7).

In a pilot analysis, we evaluated whether circulating miR-122, miR-34a and miR-16 can be used to assess the disease stage in CHC and NAFLD patients. We first performed ROC curve analyses for miR-16 and miR-122 comparing healthy controls and early disease (CHC F0-F1 and NAFLD NAS 1-4) (Figure 5A,B). Because miR-34a was under the limit of detection in controls, such analysis could not be performed for miR-34a. The area under the receiver-operator characteristic curve (AUC) values for miR-122 and miR-16 in the comparison between CHC-early and control groups were 0.90 and 0.92 respectively (Figure 5A). Both miRNAs performed better than ALT (AUC = 0.85). A similar result was obtained comparing healthy controls and NAFLD-SS with AUC values of 0.93 for miR-122, 0.96 for miR-16 and 0.91 for ALT (Figure 5B). When comparing CHC-early and CHC-advanced patients, miR-34a performed better than miR-122 (AUC = 0.84 vs AUC = 0.75) (Figure 5C). Again a similar result was obtained when comparing NAFLD-SS and NASH (AUC values of 0.75 and 0.70) (Figure 5D).

## Discussion

MicroRNA changes in the liver have been reported in disease processes such as hepatocarcinogenesis and liver fibrosis. There is however only limited information about their detection in blood and their correlation with histological disease severity in patients with chronic liver diseases. We first measured intracellular and extracellular levels of miR-122, miR-34a, miR-16 and miR-21 in HCV-infected Huh7.5 cells. miR-122 intracellular levels were not affected by HCV replication, in agreement with previous reports [8]. In contrast, miR-122 strongly accumulated in the extracellular compartment of HCV-infected cells, independently of the levels of HCV replication and production. These results suggest that there is no correlation between intracellular and extracellular levels of miR-122. Similar results were observed for miR-16 although to a lesser extent. In contrast, miR-34a was increased in both the intracellular and extracellular compartments upon HCV infection and miR-21 was only slightly changed. miRNAs are associated with cell-derived vesicles or exosomes [2] but also with protein complexes [24]. miR-122 and miR-16 in particular have been reported associated with argonaute2 complexes and independent of vesicles in plasma [24]. Future experiments should investigate the mechanisms leading to the release of these microRNAs from hepatocytic cells.

In CHC patients and in patients with NAFLD, plasma levels of miR-122 were elevated compared to healthy controls. In CHC patients, miR-122 levels correlated with fibrosis stage and inflammation activity but didn't correlate with HCV viral load. An absence of correlation between intrahepatic miR-122 and HCV RNA levels was also observed in human liver biopsies [25], [26]. In NAFLD patients, miR-122 levels also correlated with fibrosis stage and inflammation activity. An inverse correlation between intrahepatic miR-122 and fibrosis [18], [26] and reduced intrahepatic levels of miR-122 in NASH were reported [27]. Altogether, these results are consistent with a lack of correlation between miR-122 regulation in liver tissue and in serum as observed in the HCV-infected Huh7.5 cells. Circulating miR-122 levels have been reported to be elevated in patients with chronic hepatitis B viral infection [28], [29] and to correlate with liver histologic stage, inflammation grades and ALT activity [28]. Serum miR-122 levels were also higher in patients with chronic hepatitis B infection than in patients with hepatocellular carcinoma [29]. We are reporting here similar results for miR-122 in patients with chronic hepatitis C or with NAFLD, suggesting that the increase in circulating levels of miR-122 is common to chronic liver disease of all etiologies. Our study is also in agreement with a recent study reporting an increase in serum miR-122 levels in patients with chronic hepatitis C virus infection, a strong correlation between serum levels of miR-122 and serum ALT and no correlation with serum HCV RNA [30]. This last report however didn't observe a significant correlation between serum miR-122 levels and fibrosis stage. In contrast to miR-122, there is only limited information about miR-34a expression in the liver and its regulation in chronic disease. We found miR-34a levels to be largely undetectable in plasma from healthy individuals but significantly increased to detectable levels in patients with CHC or NAFLD. As for miR-122, miR-34a plasma levels correlated with fibrosis severity. Further increase of miR-34a should be evaluated in HCC as miR-34a was reported linked to disease progression from normal liver through cirrhosis to HCC [31]. While a positive correlation between intrahepatic miR-21 expression and viral load, fibrosis or serum liver transaminase levels was reported [18], we only observed a marginal increase of miR-21 in late disease stages. In contrast, miR-16 was highly elevated in early disease and slightly decreased in advanced stages. Further decrease of miR-16 should be evaluated in HCC [32].

<1?tlsb=.005w?>Non-invasive assessment of liver fibrosis is a very important goal in patients with chronic hepatitis C. miR-122 and miR-16 were more sensitive than ALT in detecting early stage disease in the studied sets of patients. In addition, miR-122 and miR-34a may have utility in assessing disease stage. Chronic viral infection of the liver is associated with insulin resistance and also associated with the development of hepatic steatosis. The severity of steatosis has been well correlated with the degree of hepatic fibrosis and the severity of insulin resistance [33], [34]. Both miR-122 and miR-34a levels correlated with fibrosis stage, inflammation activity, steatosis grade and to a lesser extent to insulin resistance and glucose levels in CHC patients. There was overall no correlation with serum lipids at the exception of a small negative correlation with high-density lipoprotein (HDL). This may result from the fact that in CHC patients, disease severity is associated with lower HDL levels [35]. Interest in applying non-invasive methods to assess liver fibrosis in patients with NAFLD has increased in recent years [36]. The overwhelming majority of persons with elevated ALT activity in the absence of viral hepatitis or excessive alcohol consumption are found to have NAFLD on liver biopsy in the US and Europe [37]. miR-34a and miR-122 may have utility in the identification of those NAFLD patients who have developed significant liver fibrosis. Interestingly, in contrast to CHC patients, mir-122 levels correlated with serum lipids in NAFLD patients. Overall, the prospect of using miR-122 and miR-34a as prognostic markers is of interest. Larger patient cohorts with distinct hepatic disease-causes and differential fibrosis states will have to be analyzed to further test the utility of circulating miR-122 and miR-34a as biomarkers for detection or monitoring of liver fibrosis.

## Supporting Information

Table S1 Patients' clinical information.
(DOC)
