# Supplementary material for: Circulating MicroRNAs in Patients with Chronic Hepatitis C and Non-Alcoholic Fatty Liver Disease
Source: PLoS One. 2011 Aug 23;6(8):e23937. doi: 10.1371/journal.pone.0023937 (PMC3160337; doi:10.1371/journal.pone.0023937)
Supplement: Table S1 — Patients' clinical information. (DOC) [file pone.0023937.s001.doc]

**Table S1. Patients’ clinical information**

| **Group I: Healthy Control (n=19)** |  |
| --- | --- |
| Age: median (range) | 51 (23-76) |
| Gender: Male/Female (%) | 63/37 |
| ALT (IU/L): median (range) | 31 (13-38) |
| **Group II: Chronic hepatitis C (CHC) (n=53)** |  |
| **Set 1 (n=18)** |  |
| Age: median (range) | 47 (40-54) |
| Gender: Male/Female (%) | 67/33 |
| ALT (IU/L): median (range) | 53 (2.6-152) |
| Viral Titer (IU/ml): median (range) | 2.8x10^4^ (3x10^3^-6x10^5^) |
| Fibrosis stage: ≤ 1/≥ 2 (%) | 47/53 |
| **Set 2 (n=35)** |  |
| Age: median (range) | 56 (31-65) |
| Gender: Male/Female (%) | 91/9 |
| ALT (IU/L): median (range) | 59 (26-394) |
| Viral Titer (IU/ml): median (range) | 4.9x10^6^ (2.4x10^4^-10x10^6^) |
| Fibrosis stage: ≤ 1/≥ 2 (%) | 60/40 |
| **Group III: Non-alcoholic fatty liver disease (NAFLD) (n=34)** |  |
| Age: median (range) | 50.5 (26-71) |
| Gender: Male/Female (%) | 85/15 |
| ALT (IU/L): median (range) | 76 (14-183) |
| NAS score: ≤ 4/≥ 5 (%) | 53/47 |
